# Decreased Left Atrial Reservoir Strain Is Associated with Adverse Outcomes in Restrictive Cardiomyopathy

**DOI:** 10.3390/jcm11144116

**Published:** 2022-07-15

**Authors:** Jadranka Stojanovska, Nevriye Topaloglu, Kana Fujikura, Behnaz Khazai, El-Sayed Ibrahim, Alex Tsodikov, Nicole M. Bhave, Theodore J. Kolias

**Affiliations:** 1 Department of Biomedical Imaging, New York University Langone Medical Center, New York, NY 10016, USA; 2 Michigan Medicine and School of Public Health, University of Michigan, Ann Arbor, MI 48109, USA; nevriye.topaloglu@yahoo.com (N.T.); behnazkhazai@icloud.com (B.K.); tsodikov@umich.edu (A.T.); nbhave@med.umich.edu (N.M.B.); tkolias@umich.edu (T.J.K.); 3 National Heart, Lung and Blood Institute, National Institute of Health, Bethesda, MD 20892, USA; kana.fujikura@nih.gov; 4 Department of Radiology, Medical College of Wisconsin, Milwaukee, WI 53226, USA; eibrahim@mcw.edu

**Keywords:** left atrial reservoir strain, restrictive cardiomyopathy, left atrial area, survival analyses, cardiac magnetic resonance

## Abstract

Background: Restrictive cardiomyopathy (RCM) places patients at high risk for adverse events. In this study, we aim to evaluate the association between left atrial function and time to adverse events such as all-cause mortality and cardiovascular hospitalizations related to RCM. Material and Methods: In this single-center study, ninety-eight patients with a clinical diagnosis of RCM were recruited from our registry: 30 women (31%); age (mean ± standard deviation) 61 ± 13 years. These patients underwent cardiac magnetic resonance (CMR) imaging from May 2007 to September 2015. Left atrial (LA) function (reservoir, contractile, and conduit strain), LA diameter and area, and left ventricular function (global longitudinal strain, ejection fraction), and volume were quantified, and the presence of late gadolinium enhancement was visually assessed. The cutoff value of the LA reservoir strain was selected based on tertile. An adjusted Cox proportional regression analysis was used to assess time to adverse outcomes with a median follow up of 49 months. Results: In our cohort, all-cause mortality was 36% (35/98). Composite events (all-cause mortality and cardiovascular hospitalizations) occurred in 56% of patients (55/98). All-cause mortality and composite events were significantly associated with a decreased LA reservoir strain (adjusted hazard ratio (aHR) = 0.957, *p* = 0.002 and aHR = 0.969, *p* = 0.008) using a stepwise elimination of imaging variables, demographics, and comorbidities. All-cause mortality and composite events were six and almost four times higher, respectively, in patients with the LA reservoir strain <15% (aHR = 5.971, *p* = 0.005, and HR = 4.252, *p* = 0.001) compared to patients with the LA reservoir strain >34%. Survival was significantly reduced in patients with an LA reservoir strain <15% (*p* = 0.008). Conclusions: The decreased LA reservoir strain is independently associated with time to adverse events in patients with RCM.

## 1. Introduction

The overall prognosis of restrictive cardiomyopathy (RCM) is poor [1,2,3], despite the clinical utility of cardiac magnetic resonance (CMR) late gadolinium enhancement (LGE), an imaging modality that has reshaped the landscape in myocardial tissue characterization as a guide to targeted therapy [4] for cardiomyopathy. RCM encompasses a spectrum of myocardial disorders with common physiology, but divergent etiologies. The diagnosis of RCM is based upon clinical, laboratory, and imaging findings [2].

The hallmark of RCM is myocardial stiffness, characterized by diastolic dysfunction that leads to impaired ventricular filling, preserved ventricular systolic function, and left atrial enlargement [2]. Left atrial (LA) enlargement/remodeling is also an emerging biomarker of adverse cardiovascular events, including mortality, which is attributed to the progression of heart failure, arrhythmias, and thrombogenicity [5]. Recent studies suggest that impaired atrial contractility predicts cardiovascular morbidity and mortality [5,6]. Furthermore, atrial fibrosis is identified as a cause of impaired atrial contractility that precedes atrial remodeling by histological analysis [5,7].

Therefore, we hypothesize that left atrial failure defined by impaired atrial function assessed by atrial strain in RCM is independently associated with all-cause mortality and cardiovascular hospitalizations. While CMR LGE imaging is clinically used in RCM patients to diagnose specific subtypes of RCM based on tissue characterization, we aimed to determine if impaired LA function in terms of reservoir and contractile strain assessed by feature-tracking is associated with all-cause mortality and cardiovascular hospitalizations independent of systemic amyloidosis, patients’ demographics, and cardiovascular disease risk factors.

## 2. Materials and Methods

### 2.1. Study Population

**Figure 1:**
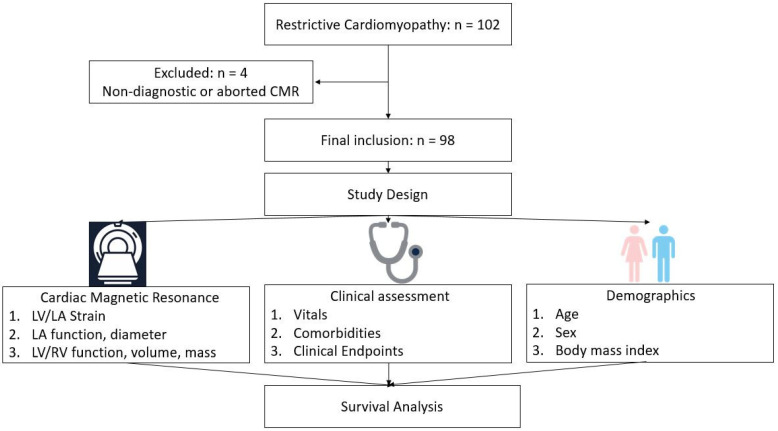
Flow chart of restrictive cardiomyopathy patients’ inclusion and study design. A total of 98 consecutive patients were included in the study after searching our CMR database for patients with clinical diagnosis of restrictive cardiomyopathy and excluding 4 patients with non-diagnostic image quality. A qualitative and quantitative assessment of cardiac MR followed by collection of demographics and comorbidities was performed. CMR = cardiac magnetic resonance; LA = left atrium; LGE = late gadolinium enhancement; LV = left ventricle; RV = right ventricle.

Institutional review board approval was obtained with a waiver of informed consent for this HIPAA-compliant retrospective cohort study. In this study, we included 98 patients with a clinical diagnosis of RCM (30 women and 68 men); age (mean ± standard deviation with same format staying in the sequel) 61 ± 13 years from our institutional CMR registry from May 2007 to September 2015 with a median follow up of 49 months. Clinically, RCM was defined based on respective guidelines which include restrictive ventricular physiology in the presence of normal or reduced diastolic ventricular volumes, systolic volumes, and overall normal ventricular wall thickness [8,9]. All patients underwent standard, clinically indicated CMR evaluation for cardiac function and LGE assessment approximately a week after initial evaluation. We included subjects with a clinical diagnosis of RCM due to primary cardiomyopathic disorder. In addition to patients with underlying multiple myeloma or systemic amyloidosis in whom the suspicion for cardiac amyloid was high, 10 patients had restrictive physiology on cardiac catheterization and three patients were suspected to have cardiac sarcoid. Exclusion criteria included: significant arrhythmias that preclude CMR acquisition which include atrial fibrillation, moderate-to-severe aortic stenosis, or mitral regurgitation based on echocardiography; the presence of a prosthetic valve; hypertrophic cardiomyopathy; or recent myocardial infarction (<6 months). In addition, 4 patients were excluded because of non-diagnostic image quality or an incomplete cardiac MR exam (Figure 1).

### 2.2. Clinical Variables and Follow up

We collected the following demographics: age, sex, heart rate, body surface area (BSA), and body mass index (BMI). Comorbidities including hypertension, diabetes, hyperlipidemia, systemic amyloidosis, and multiple myeloma were recorded. Hypertension was defined as a systolic blood pressure of at least 140 mmHg, diastolic blood pressure of at least 90 mmHg, or ongoing antihypertensive treatment [10]. Hyperlipidemia was defined as a blood cholesterol level of at least 240 mg/dL, serum triglyceride level of at least 150 mg/dL, or ongoing lipid-lowering treatment [11]. Diabetes mellitus was defined as a fasting plasma glucose level of at least 126 mg/dL and/or the current use of antidiabetic medications [12]. BMI and BSA were calculated by using patients’ weight and height recorded on the same day as the CMR [13].

### 2.3. Clinical Endpoints

For the purposes of this study, we established two endpoints. The primary endpoint was time to all-cause mortality. All-cause mortality was recorded by chart review and electronic files of death certificates. The secondary endpoint was the time to composite events, consisting of death from any cause or cardiovascular hospitalization, whichever occurred first. The reason for hospitalizations in the majority of our patients was the suspicion for heart failure followed by stroke. Information on cardiovascular hospitalizations was obtained by chart review, performed by an independent reviewer blinded to the patients’ clinical and imaging information. We used September 2018 as the end of the study for survival analysis, and patients whose survival exceeded the date were considered as type-I censored. There were no censored observations before this date.

### 2.4. Cardiac Magnetic Resonance Imaging—Acquisition

All patients were scanned in the supine position using clinical 1.5 T MRI scanners (Achieve, Philips Healthcare, Best, The Netherlands) equipped with an advanced cardiac package, radiofrequency (RF) magnetic field shimming technology and a 32-channel coil. Cardiac scans were performed to assess the presence of LGE and determine the presence of infiltrative myocardial disease. A two-dimensional (2D) segmented inversion recovery (IR) gradient-echo sequence was obtained 10 min after injection of 0.2 mols/kg of (Multihance, Bracco, Ferentino, Italy) gadolinium contrast agent with inversion time (TI) set to null normal myocardium based on a TI-scout Look-Locker sequence run immediately before the LGE sequence. The imaging protocol consisted of cine short-axis and long-axis images (steady-state with free precession (SSFP) sequence, repetition time (TR) = 4.2 ms, echo time (TE) = 1.8 ms, resolution = 1.4 × 1.4 mm^2^, slice thickness = 8 mm, flip angle = 60, readout bandwidth (BW) = 0.2) as well as 2D LGE imaging (IR sequence, TR = 6.7 ms, TE = 3.2 ms, resolution = 1.4 × 2.2 mm^2^, slice thickness = 8 mm, specific absorption rate (SAR) limit = 2 W/Kg, and inversion time (TI) = 250–350 ms based on results from the Look-Locker TI scouting sequence). Short- and long-axis planes were acquired using the same slice thickness and positions as used for cine imaging. SSFP long-axis images were acquired in two-chamber, four-chamber, and three-chamber (left ventricular (LV) outflow tract) planes, prescribed from the short-axis cine images.

### 2.5. Cardiac Magnetic Resonance Imaging—Post-Processing

**Figure 2:**
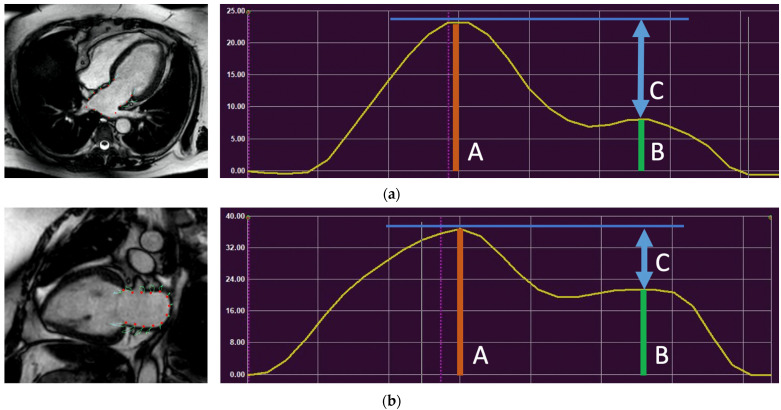
A 63-year-old man with RCM. (**a**) LA endocardial contour was traced at end-systole (i.e., LA end-diastole). The LA function was evaluated using feature-tracking analysis on four-chamber (**a**) and two-chamber (**b**) views. The zero reference was set at end-diastole. LA strain was measured at end-systole; the first peak (A-orange line) corresponds with LA reservoir strain; the second peak (B-green line) corresponds to contractile strain; and the difference between the reservoir and contractile strain (blue arrow line C) corresponds to conduit. Average LA strain was calculated as the mean value of the two-chamber and four-chamber views.

The comprehensive CMR protocol included the analysis of cardiac volume, function, mass, and myocardial contractility. The MR images were sent off-line to a research-dedicated workstation for post-processing. A visual evaluation of the short- and long-axis cine images allowed for a qualitative evaluation of the presence and distribution of chamber wall thickening. Manually tracing the epicardial and endocardial borders on the short-axis cine images at end-diastole and end-systole allowed the calculation of biventricular quantitative parameters, based on Simpson’s approximation method, including end-diastolic volume (EDV), end-systolic volume (ESV), stroke volume, ejection fraction (EF), LV mass, and LA diameter and area. In addition, the LV global longitudinal strain (GLS); left atrial (LA) strain (reservoir, conduit, and contractile); and LA emptying fraction were measured using a commercially available feature-tracking software (Suite MR 7.6 Enterprise Solution, Medis, Leiden, The Netherlands). The feature-tracking analysis was performed by a single physician trained in CMR post-processing who was blinded to the patients’ information and outcomes. On short- and long-axis cine imaging, the endocardial border of the LV was manually traced at the end-diastole, and then the endocardium was automatically tracked over the cardiac cycle. The adequacy of endocardial tracking was visually verified frame-by-frame. In cases where the endocardial tracking was not sufficient, the location of tracking was manually repositioned, and then automatic tracking was performed again. The feature-tracking analysis of the LA strain (Figure 2a,b) was performed by manually tracing the endocardial border of the LA at end-systole and end-diastole, and then the endocardium was automatically tracked over the cardiac cycle. The LA strain (reservoir, conduit, and contractile) for each patient was calculated by the average of the LA strain from the two-chamber and four-chamber views. The zero reference was set at end-diastole, and the first peak LA strain was measured at end-systole, which corresponds to the LA reservoir strain. The second peak LA strain was measured during diastole, which corresponds to the LA contractile strain. The LA conduit strain was calculated as ‘the LA reservoir strain minus the LA contractile strain’. The LA reservoir strain was divided into tertile based on the distribution. The LA reservoir strain <15% represents the lower tertile of the LA function and the LA reservoir strain between 15–34% represents the mid tertile of the LA function, whereas the LA strain >34% represents the upper tertile of the LA function. To test interobserver variability of the strain analysis, a second reader, a cardiac MR fellowship-trained physician with fourteen years of experience, repeated the LA strain analysis six month later on thirty patients.

### 2.6. Statistical Analysis

Continuous variables were summarized as mean ± SD or as median with interquartile range (IQR) where appropriate. The Kolmogorov–Smirnov test was used to assess the normality of the continuous variables [9]. Categorical variables were summarized as frequency distributions (percentages). Continuous clinical and MR imaging variables were compared according to primary and secondary endpoints using Student’s *t*-test or Wilcoxon rank sum test where appropriate. Categorical variables were compared using the chi-square test. A Cox proportional regression analysis was used to study the association between CMR measurements as well as amyloid and primary (all-cause mortality) and secondary (composite of cardiovascular hospitalization and all-cause mortality) endpoints adjusted for demographics (age, sex, body mass index, and heart rate) and comorbidities (diabetes, hypertension, multiple myeloma). Survival curves for cumulative events (primary and secondary endpoints) as a function over time for the LA reservoir strain were obtained using the Kaplan–Meier method and compared using log-rank statistics. A terile of the LA reservoir strain based on distribution with cutoffs of <15%, 15–34%, and >34% was used for graphical illustration and not derived to optimize patient outcome. A *p* value less than 0.05 was considered statistically significant. The Bonferroni correction was also applied in a multivariable analysis. Interobserver reproducibility of the LA reservoir strain obtained from the two-chamber view was assessed in thirty patients randomly selected using the Bland–Altman methodology by calculating mean difference, 95% limits of agreement, and correlation coefficient. Computations were performed using SAS/STAT software (version 9.3, SAS Institute Inc., Cary, NC, USA).

## 3. Results

### 3.1. Study Population

A total of 98 consecutive patients with RCM who underwent CMR imaging were included in the study. In total, 68 (69%) patients were men and 30 (31%) were women. The mean age of our patient population was 61 ± 13 years. In addition, 50 patients (51%) had multiple myeloma and 39 (40%) had systemic amyloidosis, 3 of which had the transthyretin type. In total, 65 (66%) patients had hypertension, and 54 (55%) patients were either hospitalized (n = 19) because of cardiovascular or cerebrovascular events (n = 6), or experienced all-cause mortality with a mean time of 53 ± 38 months. Furthermore, 35 patients (36%) experienced all-cause mortality with a mean time of 58 ± 37 months. Patients’ demographics are shown in Table 1.

### 3.2. Imaging Characteristics

In total, 46 (48%) patients demonstrated myocardial signal enhancement on the LGE images. The LGE pattern was indicative of cardiac amyloidosis in 9 out of 12 biopsy proven cardiac amyloidosis cases. In all, 48 patients (49%) had an LV ejection fraction lower than 50%. RA free wall thickening was present in seventeen patients, thirteen of which had systemic amyloidosis. The LA wall did not demonstrate wall thickening (Table 2). We demonstrated an excellent interobserver repeatability of the LA strain analysis derived from the two-chamber view between the two readers (Bland–Altman test results: mean = −0.02, standard deviation (SD) = 0.99, minimum = −1.65, maximum = 2.80, where all measurement differences lay within the 95% limits of agreement; *p* value = 0.108).

### 3.3. Time to All-Cause Mortality

**Figure 3:**
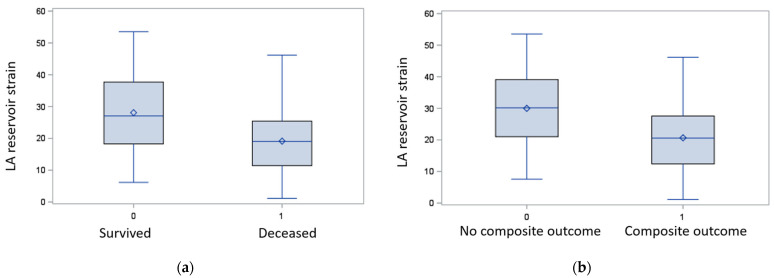
Boxplots of LA reservoir strain in RCM who experienced death (**a**) and composite events of death and cardiovascular hospitalizations (**b**). (**a**) LA reservoir strain is significantly decreased in patients who died (labeled 1) (*p* = 0.002). (**b**) LA reservoir strain is significantly decreased in patients who experienced the composite endpoint of death or cardiovascular hospitalizations (labeled 1) (*p* = 0.0008). LA = left atrium; RCM = restrictive cardiomyopathy.

**Figure 4:**
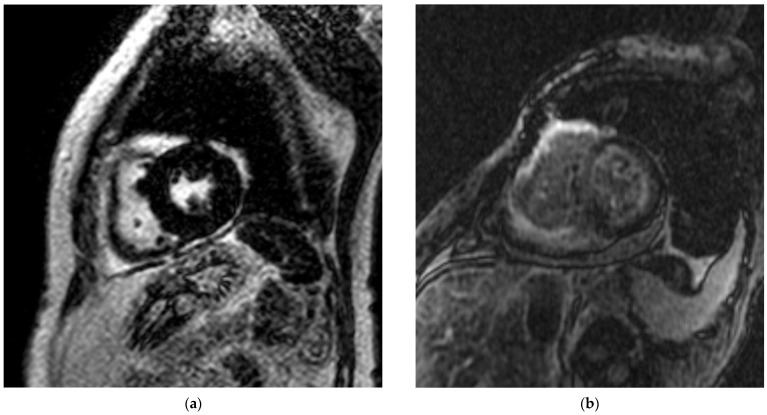
Representative case of RCM patient who died. (**a**) A 72-year-old female with multiple myeloma and hypertension referred for cardiac magnetic resonance to evaluate infiltrative cause of restrictive cardiomyopathy. Late gadolinium enhancement image does not show positive signal enhancement of the left ventricular myocardium. Patient’s left ventricular systolic function was 45% and LA reservoir strain was 14%. Patient expired six months after the CMR was performed. (**b**) A 62-year-old woman with multiple myeloma and hypertension referred for CMR to evaluate for underlying cause of restrictive cardiomyopathy. Late gadolinium enhancement image showed positive global subendocardial late gadolinium enhancement of the left and right ventricular myocardium. Patient’s left ventricular systolic function was 45% and left atrial function by LA reservoir strain was 14%. Patient expired two weeks after this CMR. CMR = cardiac magnetic resonance; LA = left atrium.

**Figure 5:**
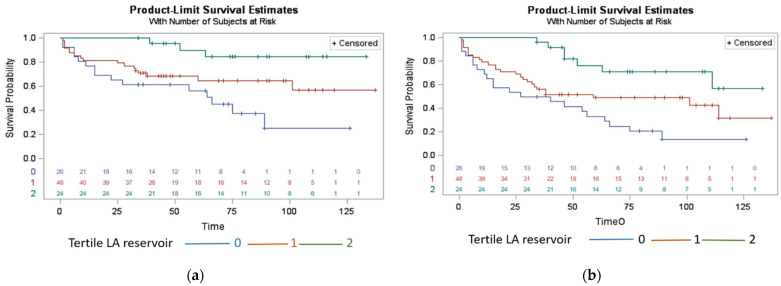
Kaplan–Meier plots of RCM patients’ survival and composite events by using mild, moderate, and severe LA functional impairment based on left atrial reservoir strain. (**a**) Patients in the lowest tertile with LA reservoir strain of <15% experienced death at earlier time points than patients with left atrial function >15% (*p* = 0.008). (**b**) Patients in the lowest tertile with LA reservoir strain of <15% experienced composite events of death and cardiovascular hospitalizations at earlier time points than patients with left atrial function >15% (*p* = 0.002). Left atrium = LA.

In total, 35 (36%) patients experienced all-cause mortality within 58 ± 37 (mean ± SD) months. Among them, 23 patients (67%) had systemic amyloidosis demonstrating a significantly higher prevalence when compared to patients who survived (*p* = 0.0002). The patients who did not survive were significantly older (mean age of 66 ± 11 years) compared to patients who survived (mean age of 59 ± 13 years) (*p* = 0.017). A decreased LA reservoir strain (Figure 3a) and LA contractile strain were the only imaging variables significantly associated with all-cause mortality (hazard ratio (HR) = 0.952, 95% confidence interval (CI) = 0.924–0.981, *p* = 0.001 and HR = 0.936, CI = (0.887–0.987), *p* = 0.015, respectively) in the univariate regression analysis. All-cause mortality in RCM patients was significantly associated only with the decreased LA reservoir strain in the adjusted analysis (aHR = 0.902, CI = (0.851–0.957) *p* = 0.0006) (Table 3A, Figure 4b). The LA contractile strain and LV GLS were not associated with all-cause mortality (aHR = 0.903, *p* = 0.035 and aHR = 1.032, *p* = 0.240, respectively). In addition, neither LV EF (aHR = 1.030, *p* = 0.091) nor LA diameter (aHR = 1.038, *p* = 0.332) were associated with all-cause mortality in the multivariable analysis. Furthermore, while systemic amyloidosis was associated with all-cause mortality in the univariate analysis (aHR = 3.374, *p* = 0.0006), this association was attenuated in the analysis adjusted for demographics and comorbidities (aHR = 2.122, *p* = 0.087). Survival period was significantly shorter in patients with the LA reservoir strain <15% (*p* = 0.008) (Figure 5a). The presence of LGE was not associated with time to all-cause mortality (*p* = 0.530) (Table 3A).

### 3.4. Time to Composite Events (All-Cause Mortality and Cardiovascular Hospitalizations)

In total, 55 patients (56%) experienced composite events with a mean time of 52 ± 38 months. Patients who experienced adverse outcomes were significantly older than patients who did not experience adverse outcomes (mean age 65 ± 11 versus 57 ± 13 years, *p* = 0.002, Table 1). No significant differences were observed between men and women, or between patients with and without cardiovascular risk factors such as diabetes, hyperlipidemia, hypertension, systemic amyloid, and obesity between the two groups (Table 1).

There was a significant association between most CMR imaging variables and adverse composite events (Table 3B) in the univariate analysis. After adjusting for imaging variables, demographics, and comorbidities, composite events were significantly associated only with (Table 3B) the decreased LA reservoir strain (adjusted hazard ratio aHR = 0.923, (0.879–0.969), *p* = 0.0012 (Figure 3b and Figure 4a,b). The LA contractile strain was not associated with composite events in the multivariate analysis after Bonferroni correction (aHR = 0.912, *p* = 0.016). In addition, neither the LV EF (aHR = 1.004, *p* = 0.751) nor LA diameter (aHR = 1.027, *p* = 0.331) were associated with composite events in the multivariable analysis. Furthermore, the systemic amyloidosis was not associated with composite events neither in the univariate analysis (aHR = 1.652, *p* = 0.065), nor in the analysis adjusted for demographics and comorbidities (aHR = 1.113, *p* = 0.753). The time to composite event period was significantly shorter in patients with a decreased LA reservoir strain < 15% (*p* = 0.002) (Figure 5b).

## 4. Discussion

### 4.1. Main Findings

Our main findings indicate that the impaired LA reservoir strain is independently associated with time to all-cause mortality and composite events in RCM patients. The time to composite event and all-cause mortality period was significantly shorter in patients with a decreased LA reservoir strain < 15%. RCM patients who experienced both outcomes were older; however, age did not decrease the significance of the severely decreased LA reservoir strain. In addition, systemic amyloidosis attenuated its association with all-cause mortality in the multivariable analysis.

### 4.2. All-Cause Mortality and Left Atrial Function

Left atrial remodeling/cardiomyopathy as a consequence of underlying myocardial disease could be a superior biomarker to underlying myocardial disease for risk stratifying of patients with RCM. LA dilation defined by LA size or volume as a measure of LA remodeling has been used as a prognostic marker of adverse events in other patient populations than RCM. For example, LA dilation is an important prognostic marker of reduced exercise capacity; chronicity of elevated LV filling pressures; and adverse cardiovascular outcomes, including stroke, atrial fibrillation, congestive heart failure; and mortality in populations with pre-existing cardiovascular diseases, such as post-myocardial infarction or with LV dysfunction [14,15,16,17,18,19,20]. In addition, the LA function assessed by strain echocardiography has also been used as a prognostic marker of cardiovascular events. For example, Cameli et al. [21] demonstrated a strong independent association between the decrease of LA myocardial deformation assessed by speckle tracking echocardiography and cardiovascular events. Furthermore, this study underlines the superiority of LA myocardial deformation to LA EF and LA dilation for the assessment of cardiovascular events [21]. While a number of conventional echocardiographic parameters were analyzed by Singh et al. [22], only the peak LA strain changed progressively and remained significantly different between all diastolic dysfunction grades. Studies [23,24] showed that the abnormal LA function, using strain echocardiography criteria, was identified in a significant number of cardiac amyloidosis patients where the mean peak LA strain rate was lower in these patients. LA dysfunction was observed in amyloid patients without any other echocardiographic features of cardiac involvement and may provide additional prognostic information [24,25]. In another study [26], altered LA mechanical contractility by CMR in patients with or at risk of diastolic dysfunction from hypertension provided a unique independent prognostic value to adverse cardiovascular events. Furthermore, echocardiographic strain analyses detected LA myocardial dysfunction in Fabry patients, even when conventional cardiac measurements were normal and were associated with a worse symptomatic status in Fabry patients [27]. While all these studies demonstrated the association of LA remodeling with cardiovascular events in other patient populations than RCM, only a study that was performed on ninety-two cats [3] showed that LA dilation was associated with mortality in RCM. The findings from that study agree with those from our study that LA remodeling is crucial in determining disease prognosis.

### 4.3. Time to Composite Events and Left Atrial Function

Assomull et al. [28] have reported mid-wall LGE as a predictor of the combined endpoint of all-cause death and hospitalizations in 101 patients with dilated cardiomyopathy (DCM). In our study, we did not identify LGE to be significantly associated with the composite events in our patient population of RCM. However, we identified that only the LA reservoir strain is associated with composite events of all-cause mortality and cardiovascular hospitalizations independent of LA diameter, LV ejection fraction, and LV GLS, which are both markers considered to reflect LV myocardial stiffness. It should be noted that Assomull et al. [28] used a DCM patient population with moderately depressed LV EF. While our population demonstrated mildly depressed LV EF, the hallmark of RCM is diastolic dysfunction and subsequent atrial remodeling/failure [29].

### 4.4. LGE and Endpoints

LGE is a validated and clinically useful imaging technique to diagnose myocardial diseases and predict major adverse cardiac events in patients with dilated cardiomyopathy (DCM) [30]. The presence of myocardial scar in patients with DCM may serve as a focus for arrhythmias. Furthermore, the role of LGE is to define RCM subtypes based on the pattern of LGE that is suggestive of an underlying myocardial disease. For example, in a study of 47 consecutive patients with biopsy proven cardiac amyloidosis, only LGE was associated with the diagnosis of amyloidosis and the ascertained all-cause mortality [31]. However, in our study, LGE, a standard CMR sequence for myocardial tissue characterization, was neither associated with all-cause mortality nor with the composite events. Only 20 patients had biopsy proven cardiac amyloidosis in our cohort, and 13 of them had positive LGE. However, in a more recent study by Pan et al. native T1 mapping and the extracellular volume map as a quantitative measure of diffuse myocardial process demonstrated superior diagnostic and prognostic performance to LGE in patients with amyloidosis [32]. Our findings and the findings from the recent study demonstrate that other CMR indices as markers to earlier myocardial structural changes, such as an increase in extracellular volume, than LGE can be used to determine the underlying myocardial disease and optimize management strategies in patients with suspected infiltrative myocardial disease.

### 4.5. Prior Studies

The study involving cats with RCM [3] demonstrated the importance of the evaluation of LA remodeling as a marker of disease prognosis. While LA dilation demonstrates LA remodeling and failure, the LA reservoir strain as a measure of LA function represents an early stage of LA cardiomyopathy. Our study showed that only the depressed LA reservoir strain is independently associated with all-cause mortality and composite events. In addition, the behavior of RCM may not be translated from animals to humans.

### 4.6. Study Limitation

This was a single-center, retrospective analysis of LA function using feature-tracking in patients with RCM as part of the institutional CMR registry. Some clinical parameters such as New York Heart Association classification, N-terminal (NT) pro-B natriuretic peptide, and diastolic parameters were not available and therefore represent a limitation. A relatively small study population and consequently low event rate make the extensive multivariable analysis challenging; however, we used the Bonferroni correction to adjust for the significance level [14]. A larger scale multi-center prospective study is needed to confirm our results. Although a feature-tracking analysis depends on analyzing fewer features compared to a conventional tagging analysis [26], tagging is not applicable for analyzing thin-walled LA; therefore, CMR feature-tracking is an optimal tool for strain analysis in the atria. All clinical information was identified in our database. Native T1 mapping and extracellular volume map were not part of our routine CMR examination and therefore not acquired. Left atrial volume was not quantified; however, we quantified LA area and diameter. All measurements were performed by a single reader and only thirty patients were re-measured to assess inter-rater variability. This is reflective of the real time clinical days when one cardiac MR imaging physician reads the studies and performs the measurements. However, given the availability of automated contouring, this may not pose any limitation in the future [15].

### 4.7. Future Direction

While future prospective multi-center trials are necessary to assess the utility of the LA reservoir strain independent of LGE and T1 mapping, this study poses an excellent foundation to future well-designed studies that will guide the clinical implementation and bring this modality into everyday clinical practice to manage patients with a restrictive physiology. The automated contouring of the cardiac chambers available with most cardiac software brings this methodology into the clinical realm while maintain reporting efficiency. This measurement may prove beneficial in patients with restrictive physiology, in whom gadolinium administration may not be recommended [14].

## 5. Conclusions

The decreased LA reservoir strain was associated with time to adverse events in patients with RCM independent of CMR measures of LV myocardial stiffness. While LGE remains valuable for tissue characterization and diagnosing myocardial diseases, in our study, it was associated with neither all-cause mortality nor the composite events. In addition, the presence of systemic amyloidosis did not attenuate the significance of the association between the LA reservoir strain and adverse events. An LA strain analysis is a valuable CMR methodology to risk stratify RCM patients without the need for gadolinium administration.

## Figures and Tables

**Table 1:** Patients’ demographics stratified by two endpoints: time to composite events and survival.

	Composite Events			Survival			All
Baseline Characteristics	Events (n = 55)	No Events (n = 43)	*p*-Value	Non-Survived (n = 35)	Survived (n = 63)	*p*-Value	
Age (years)	65 ± 11	57 ± 13	0.002 *	66 ± 11	59 ± 13	0.017 *	61 ± 13
Sex (female)	19 (34%)	11 (25%)	0.383	13 (26%)	17 (34%)	0.369	30 (31%)
Diabetes n (%)	22 (40%)	28 (40%)	0.285	14 (28%)	20 (40%)	0.507	50 (51%)
Hyperlipidemia n (%)	17 (31%)	19 (44%)	0.208	10 (44%)	26 (31)	0.275	36 (37$)
Hypertension n (%)	39 (71%)	26 (60%)	0.291	24 (60%)	41 (71%)	0.824	65 (66%)
Systemic Amyloid n (%)	26 (47%)	13 (30%)	0.100	23 (30%)	16 (47%)	0.0002 *	39 (40%)
Multiple Myeloma n (%)	32 (58%)	18 (42%)	0.153	24 (42%)	26 (58%)	0.011 *	50 (51%)
BMI (kg/m^2^)	29 ± 7	30 ± 6	0.193	28 ± 7	30 ± 6	0.114	29 ± 7
BSA (cm/m^2^)	2.00 ± 0.3	2.07 ± 0.2	0.079	1.97 ± 0.3	2.07 ± 0.2	0.036 *	2 ± 0.2

Note: BMI = body mass index, BSA = body surface area, kg = kilogram, m^2^ = meter square, n = number; % = percent; * statistical significance *p* < 0.05.

**Table 2:** Imaging characteristics stratified by the two endpoints: time to composite events and survival.

	Composite Outcome			Survival Outcome			All
CMR Variables	Events (n = 55)	No Events (n = 43)	*p*-Value	Non-Survived (n = 35)	Survived (n = 63)	*p*-Value	
LGE	29 (53%)	17 (39%)	0.224	19 (53%)	27 (39%)	0.298	48 (49)
LA reservoir strain (%)	21 ± 11	30 ± 12	0.0008 *	19 ± 11	28 ± 12	0.002 *	25 ± 12
LA contractile strain (%)	11 ± 7	15 ± 6	0.002 *	11 ± 8	14 ± 8	0.007 *	13 ± 7
LA EDV (mL)	56 ± 36	40 ± 23	0.029 *	56 ± 36	44 ± 25	0.184	49 ± 31
LA EF (%)	42 ± 21	53 ± 15	0.011 *	41 ± 22	50 ± 17	0.054	47 ± 19
LV EDV (indexed)	92 ± 30	92 ± 39	0.529	88 ± 30	94 ± 36	0.479	95 ± 35
LV EF (%)	48 ± 16	50 ± 15	0.458	48 ± 17	49 ± 15	0.988	49 ± 15
RV EF (%)	48 ± 15	48 ± 13	0.924	45 ± 16	49 ± 12	0.449	49 ± 13
RV EDV (indexed)	89 ± 30	79 ± 23	0.147	90 ± 34	81 ± 22	0.323	82 ± 27
LA diameter (mm)	42 ± 7	39 ± 6	0.057	42 ± 7	41 ± 7	0.309	41 ± 7
LA area (mm)	25 ± 7	22 ± 4	0.045 *	25 ± 7	23 ± 5	0.075	23 ± 6
LV mass (indexed)	84 ± 36	62 ± 22	0.0006 *	79 ± 31	71 ± 33	0.051	74 ± 32
LV GLS (%)	−18 ± 9	−20 ± 7	0.163	−18 ± 10	−20 ± 7	0.391	−19 ± 8

EF = ejection fraction, EDV = end-diastolic volume, GLS = global longitudinal strain, mm = millimeter, mm^3^ = millimeter cube, mL = milliliter, LA = left atrium, LGE = late gadolinium enhancement, LV = left ventricle; (%) = percent, RV = right ventricle; LA EF = left atrial emptying fraction; * statistical significance *p* < 0.05.

**Table 3:** (**A**) Cox proportional regression analysis of cardiac magnetic resonance imaging variables and survival. (**B**) Cox proportional regression analysis of cardiac magnetic resonance imaging variables and composite events.

(A)								
Variable	Univariate Regression Analysis				Adjusted Regression Analysis *			
	HR	95% CI		*p*-Value	HR	95% CI		*p*-Value
LA reservoir strain	0.952	0.924	0.981	0.001 **	0.902	0.851	0.957	0.0006 **
LA contractile strain	0.936	0.887	0.987	0.015 **	0.903	0.821	0.993	0.035
LA diameter	1.030	0.982	1.081	0.220	1.022	0.961	1.088	0.482
LA EF	0.979	0.961	0.997	0.023 **	1.043	1.010	1.076	0.010
LGE	1.651	0.845	3.224	0.142	1.596	0.754	3.377	0.222
LV GLS	1.024	0.989	1.061	0.179	1.008	0.959	1.060	0.760
LV EF	0.996	0.975	1.017	0.684	1.030	0.995	1.065	0.091
Systemic amyloidosis	3.374	1.678	6.784	0.0006 **	2.122	0.896	5.025	0.087
**(B)**								
**Variable**	**Univariate Regression Analysis**				Adjusted Regression Analysis *			
	**HR**	**95% CI**		***p*-Value**	**HR**	**95% CI**		***p*-Value**
LA reservoir strain	0.960	0.938	0.982	0.0006 **	0.925	0.879	0.969	0.001 **
LA contractile strain	0.939	0.900	0.981	0.004 **	0.912	0.846	0.983	0.016
LA diameter	1.042	1.002	1.083	0.040 **	1.027	0.973	1.085	0.335
LA EF	0.980	0.965	0.995	0.009 **	1.037	1.006	1.068	0.017
LGE	1.684	0.984	2.881	0.057 **	1.549	0.870	2.756	0.224
LV GLS	1.021	0.992	1.052	0.155	1.013	0.968	1.060	0.580
LV EF	0.993	0.976	1.010	0.415	1.004	0.978	1.051	0.753
Systemic amyloidosis	1.652	0.968	2.818	0.065	1.113	0.570	1.031	2.174

Note: * Continuous variables adjusted for age, sex, body mass index, heart rate, diabetes, hypertension, and multiple myeloma. LA reservoir strain and contractile strain included all imaging variables, demographics, and comorbidities in the model. Significance level with Bonferroni correction at *p* < 0.003 **. CI = confidence interval, EF = ejection fraction, GLS = global longitudinal strain, HR = hazard ratio, LA = left atrium, LGE = late gadolinium enhancement, LV = left ventricle.

## Data Availability

Data are not publicly available and can be made available upon reasonable request.
